# Aspirin counteracts cancer stem cell features, desmoplasia and gemcitabine resistance in pancreatic cancer

**DOI:** 10.18632/oncotarget.3171

**Published:** 2015-02-05

**Authors:** Yiyao Zhang, Li Liu, Pei Fan, Nathalie Bauer, Jury Gladkich, Eduard Ryschich, Alexandr V. Bazhin, Nathalia A. Giese, Oliver Strobel, Thilo Hackert, Ulf Hinz, Wolfgang Gross, Franco Fortunato, Ingrid Herr

**Affiliations:** ^1^ Molecular OncoSurgery, University of Heidelberg and German Cancer Research Center (DKFZ), Heidelberg, Germany; ^2^ Section Surgical Research, University of Heidelberg, Heidelberg, Germany; ^3^ Department of General, Visceral and Transplantation Surgery, University of Heidelberg, Heidelberg, Germany; ^4^ Gastrointestinal Surgery, Zhongshan Hospital of Xiamen University, Xiamen, China

**Keywords:** Pancreatic cancer, Cancer stem cells, Novel therapeutics

## Abstract

Pancreatic ductal adenocarcinoma (PDA) is characterized by an extremely poor prognosis. An inflammatory microenvironment triggers the pronounced desmoplasia, the selection of cancer stem-like cells (CSCs) and therapy resistance. The anti-inflammatory drug aspirin is suggested to lower the risk for PDA and to improve the treatment, although available results are conflicting and the effect of aspirin to CSC characteristics and desmoplasia in PDA has not yet been investigated. We characterized the influence of aspirin on CSC features, stromal reactions and gemcitabine resistance. Four established and 3 primary PDA cell lines, non-malignant cells, 3 patient tumor-derived CSC-enriched spheroidal cultures and tissues from patients who did or did not receive aspirin before surgery were analyzed using MTT assays, flow cytometry, colony and spheroid formation assays, Western blot analysis, antibody protein arrays, electrophoretic mobility shift assays (EMSAs), immunohistochemistry and *in vivo* xenotransplantation. Aspirin significantly induced apoptosis and reduced the viability, self-renewal potential, and expression of proteins involved in inflammation and stem cell signaling. Aspirin also reduced the growth and invasion of tumors *in vivo*, and it significantly prolonged the survival of mice with orthotopic pancreatic xenografts in combination with gemcitabine. This was associated with a decreased expression of markers for progression, inflammation and desmoplasia. These findings were confirmed in tissue samples obtained from patients who had or had not taken aspirin before surgery. Importantly, aspirin sensitized cells that were resistant to gemcitabine and thereby enhanced the therapeutic efficacy. Aspirin showed no obvious toxic effects on normal cells, chick embryos or mice. These results highlight aspirin as an effective, inexpensive and well-tolerated co-treatment to target inflammation, desmoplasia and CSC features PDA.

## INTRODUCTION

Pancreatic ductal adenocarcinoma (PDA) is one of the most aggressive malignancies and is typically diagnosed at an advanced state, with extensive local invasion, early systemic dissemination and marked resistance to chemo- and radiotherapy [1]. The poor response to chemotherapy is thought to be due in part to the fibrotic nature of PDA that prevents even small therapeutic molecules from entering and perfusing the extremely dense extracellular matrix (ECM) [2]. This desmoplasia is derived from pancreatic stellate cells that are activated by inflammation and in turn proliferate and produce collagens, fibronectin and other ECM components, which participate in cancer initiation, progression and metastasis [3–6].

Increased expression of cancer stem cell (CSC) markers, including c-Met, CD133, CD44, EpCAM (also known as ESA), ALDH1, Nanog, Oct4 and SOX2, has been identified in PDA [7, 8]. According to the CSC hypothesis, stem-like cancer cells exhibit properties of normal stem cells, such as chemotherapy resistance, high DNA repair capacity, apoptosis resistance and self-renewal potential, with the latter being characterized by the ability to form spheroids and colonies and to recapitulate a tumor after xenotransplantation [9]. Furthermore, recent studies indicate a role for aberrantly high NF-κB activity in providing signals that maintain mammary CSCs [10] and demonstrate that constitutively enhanced NF-κB binding of the subunits c-Rel and p65 confers CSC features in highly aggressive PDA cells [11].

Acetylsalicylic acid (aspirin) is a potent inhibitor of NF-κB signaling [12, 13], suggesting that this drug may contribute to the elimination of pancreatic CSCs; however, to our knowledge, this issue has not yet been studied. Aspirin is based on salicylic acid, the active ingredient from willow bark, which has been used since antiquity for the treatment of headaches, pain and fever [14]. In 1897, the derivate acetylsalicylic acid was synthesized by Felix Hoffman, and it caused less digestive distress than pure salicylic acid [14]. The most understood molecular effect of aspirin is the inhibition of COX-2, and aspirin is widely prescribed for the prevention of cardiovascular events due do its blood-thinning properties [15]. However, an increasing body of evidence supports a role for aspirin in cancer prevention and in the reduction of metastasis and mortality, especially for colon cancer [16, 17]. In PDA, a decreased cancer risk associated with aspirin was observed in a prospective study conducted from 1992 through 1999 in the US among 28,283 post-menopausal women; among 80 incident cases of pancreatic cancer, the authors stated that 43% of pancreatic cancer cases among non-users of aspirin might have been prevented by the daily intake of aspirin [18]. An analysis of individual patient data from 8 randomized clinical trials reported that long-term daily aspirin use significantly reduced deaths due to PDA, with the greatest benefit obtained after 5 years of aspirin use [17]. These results were confirmed with transgenic mice, where aspirin significantly inhibited the progression of pancreatic intraepithelial neoplasia (PanIN) and tumor formation [19], and together with gemcitabine, extended survival [20]. However, data gathered as part of the Nurses´ Health Study involving almost 90,000 women in the US indicated that women who took 2 or more standard aspirin tablets per week for more than 20 years had a 59% increased risk of PDA compared with women who rarely, or never, used the medication [21].

To shed new light on the still conflicting role of aspirin in PDA, we evaluated the efficacy of this drug in the elimination of CSC features. We found that aspirin inhibited the potential for self-renewal, stem cell signaling, tumor growth and invasion along with inhibition of markers for inflammation and it sensitized PDA to gemcitabine-mediated cytotoxicity *in vitro* and *in vivo*. Most importantly, aspirin diminished the levels of ECM components and had no toxic side effects.

## RESULTS

### Aspirin increases the therapeutic efficacy of gemcitabine

**Figure 1:**
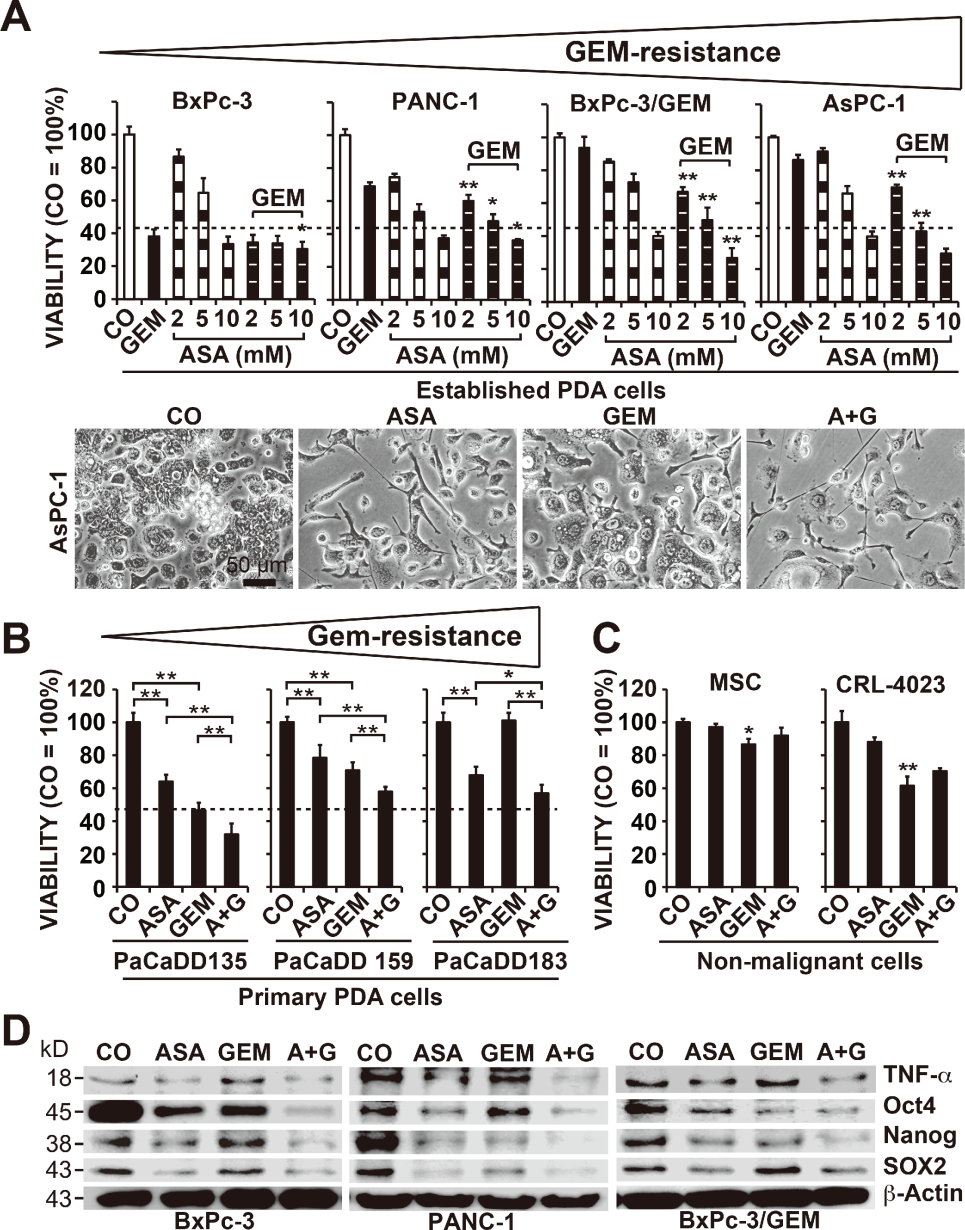
Aspirin overcomes gemcitabine resistance and alters the expression of reprogramming factors **(A)** The human established PDA cell lines BxPc-3, PANC-1, BxPC-3/GEM and AsPC-1 were left untreated (CO) or were treated with 2, 5, or 10 mM aspirin (ASA), 50 nM gemcitabine (GEM), or both together, and the cellular viability was measured 72 h later with an MTT assay. The gemcitabine resistance of the cells is indicated by a triangle above the diagrams. The morphology of AsPC-1 cells 72 h after treatment was documented by microscopy at 200× magnification. Representative pictures are shown, and the bar indicates 50 μm. **(B)** The primary, low-passage human PDA cell lines PaCaDD183, PaCaDD159 and PaCaDD135 or **(C)** human MSCs and non-malignant, immortalized human pancreatic ductal cells (CRL-4023) were treated and analyzed as described above. **(D)** BxPc-3, PANC-1 and BxPc-3/GEM cells were treated as described above, and after 48 h, expression of the aspirin target and pro-inflammatory factor TNF-α and expression of the re-programming factors Oct-4, Nanog and SOX2 were measured by Western blot analysis. β-Actin served as a control. The data are presented as means ± SD (**P < 0.01, *P < 0.05).

To evaluate the effect of aspirin on gemcitabine responsiveness, we examined 4 established PDA cell lines with different grades of aggressiveness [11, 22] and gemcitabine resistance (Figure 1A). The cells were treated with gemcitabine or 3 different aspirin concentrations, or a combination thereof. Viability, cell morphology and apoptosis were determined 72 h after treatment according to the MTT assay, microscopy, and staining with AnnexinV-FITC followed by flow cytometry, respectively (Figure 1A, Figure S1A, S1B). Whereas gemcitabine was effective in sensitive BxPc-3 cells, as expected, it was minimally effective in median resistant PANC-1 cells and non-effective in the totally resistant BxPc-3/GEM and AsPC-1 cells. In contrast, aspirin at doses of 5 and 10 mM was effective in all cell lines, and aspirin treatment significantly increased the efficacy of gemcitabine upon combination. These results were confirmed in the primary, low-passage, human PDA cell lines PaCaDD135 (gemcitabine-sensitive), PaCaDD159 (median gemcitabine-resistant) and PaCaDD183 (totally gemcitabine-resistant), which were in passage 5 (Figure 1B). Most importantly, aspirin did not significantly affect the viability of non-malignant human primary MSCs or immortalized human pancreatic ductal CRL-4023 cells, in contrast to gemcitabine, which significantly reduced the viability of MSCs and CRL-4023 cells (Figure 1C).

To assess the mechanism responsible for these results, we examined the expression of the aspirin target TNF-α, a pro-inflammatory cytokine, which induces NF-κB activity [13, 23], and the self-renewal and stem cell markers Oct4, Nanog and SOX2 [7, 8] by Western blot analysis. Treatment of cells with aspirin for 48 h significantly reduced the expression of these proteins, which was further decreased by combination treatment with gemcitabine (Figure 1D). These results were confirmed using the Human Pluripotent Stem Cell Antibody Protein Array, which demonstrated significant aspirin-induced inhibition of Oct-3/4, SOX2 and Nanog protein levels in PANC-1 cells, along with the induction of E-Cadherin and inhibition of α-Fetoprotein, PDX-1, SOX17, Otx2, TP63, Goosecoid, VEGFR-2, HCG, GATA-4, FoxA2 and Snail (Figure S1C). Moreover, aspirin inhibited the binding activity of NF–κB (Figure S2A), whose activity has been linked to CSC features [11]. In contrast, gemcitabine reduced the NF-κB binding activity only in low aggressive BxPc-3 cells and had no effect, or led to increased NF-κB binding, in more aggressive cells. Remarkably, gemcitabine-induced NF-κB activity was reduced to below basal levels in combination with aspirin. Furthermore, expression of the NF-κB subunits cRel, p65 and p52 was inhibited by aspirin, with increased effects upon combination treatment, as determined by Western blot analysis (Figure S2B).

**Figure 2:**
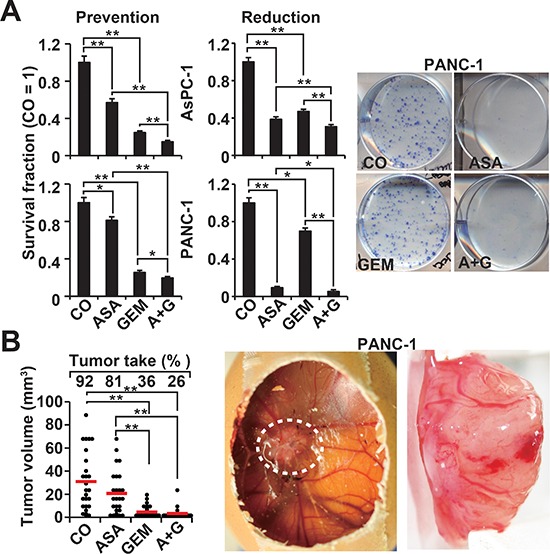
Aspirin inhibits the potential for self-renewal and enhances gemcitabine efficacy **(A)** AsPC-1 and PANC-1 cells were treated as described in Figure 1A, followed by re-plating of viable cells 48 h later at a low density (AsPC-1: 400 cells/well; PANC-1: 800 cells/well) in 6-well plates. After two weeks, colonies containing more than 50 cells were counted using a dissecting microscope. The number of surviving colonies in the control was set to 1, and the survival fraction is presented on the left (Prevention). The diagrams on the left (Reduction) are based on experiments in which the cells were seeded at a low density, and 7 days later, after colony formation, the cells were treated as described. After an additional 5 days, clonogenic survival was analyzed as described above. Representative photographs of fixed and Coomassie-stained PANC-1 colonies in the prevention experiment are presented on the left. **(B)** PANC-1 cells were treated as described, and 48 h later, the cells were transplanted onto the CAM of fertilized chick eggs at day 8 of embryonic development. At day 17, the percentage of engraftment (tumor take) and the tumor volumes were evaluated. Photographs of a developed xenograft on the CAM (white, dotted line) and a resected xenograft tumor are shown on the right. The volumes of individual tumors (dots) and the means of each group (bar) ± SD (**P < 0.01, *P < 0.05) are shown.

**Figure 3:**
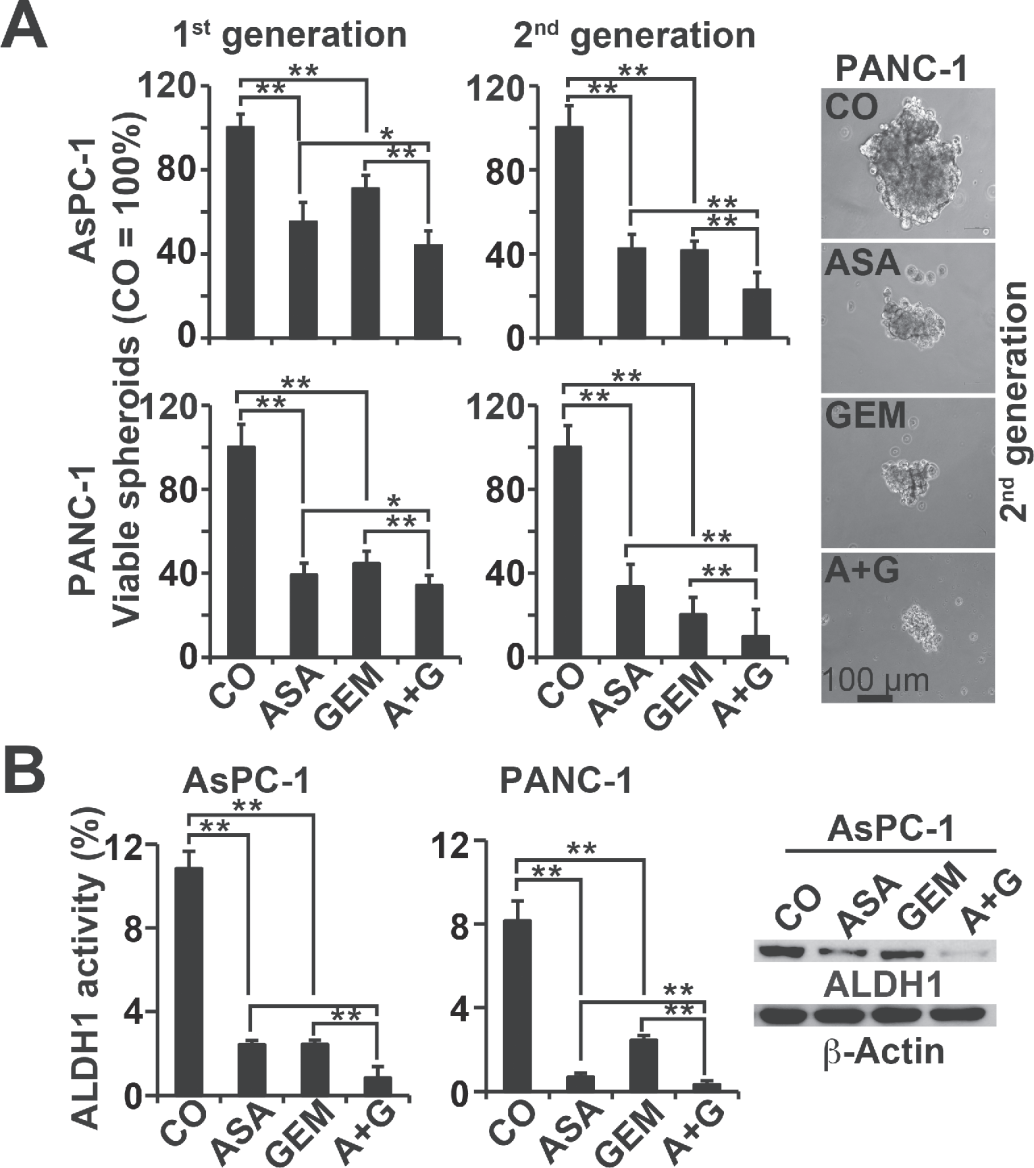
Aspirin inhibits spheroid formation and ALDH1 activity and enhances gemcitabine efficacy **(A)** AsPC-1 and PANC-1 cells were seeded at a clonal density (1.0–2.0 × 10^3^ cells/mL) in ultra-low attachment plates with serum-free, but growth factor-containing, medium for spheroid formation. Forty-eight hours later, the cells were treated as described, and then 72 h later, the percentage of viable spheroids was determined (1st Gen). The number of spheroids in the control was set to 100%. Thereafter, 1^st^ Gen spheroids were dissociated to single cells, and equal numbers of live cells were re-plated at a concentration of 1.0–2.0 × 10^3^ cells/mL. Upon spheroid formation 72 h later, the cells were photographed at 100× magnification (left images) and quantified as described above (2nd Gen). The bar indicates 100 μm. **(B)** AsPC-1 and PANC-1 cells were treated as described above, and 48 h later, ALDH1 activity was analysed using a substrate assay and flow cytometry. Western blot analysis of ALDH1 protein expression in AsPC-1 cells 48 h after treatment is shown on the left. The data are presented as means ± SD (**P < 0.01, *P < 0.05).

To study the influence of aspirin on the potential for self-renewal, AsPC-1 and PANC-1 cells were treated and then seeded for colony formation. After two weeks, aspirin and gemcitabine alone significantly inhibited colony formation in the more aggressive cells, and combination treatment exhibited an even stronger effect (Figure 2A, Prevention, Figure S2C). In a second approach, full-grown colonies were treated with the result, that aspirin reduced the survival fraction of colonies much stronger than in the prevention experiment (Figure 2A, Reduction). This variance between the prevention and protection assays may be expected, because the experimental settings differ. To assess the effect on tumor growth, we used xenotransplantation to fertilized chick eggs, which is an *in vivo* replacement method for mouse studies. Chick embryos are naturally immunodeficient and immunocompetence in birds develops only after hatching [24]. Thus, the situation resembles immunocompromised mice. Most importantly, the chorioallantoismembrane (CAM) of the chick embryo is non-innervated and therefore the chick embryo does not feel pain by CAM transplantation and tumor growth on the CAM [25–27], as mice do by the subcutaneous or orthotopic transplantation procedure and subsequent tumor growth. PANC-1 cells were treated *in vitro* and then transplanted to the CAM of fertilized chick eggs (Figure 2B). Both aspirin and gemcitabine reduced the engraftment efficacy (percentage of tumor take) and the tumor volumes, and the combination treatment led to the most pronounced effect. These results were confirmed by treatment of AsPC-1- and PANC-1-derived spheroids and the measurement of viable spheroids after treatment (Figure 3A, 1st generation). Aspirin and gemcitabine both significantly reduced the percentage of spheroids, but both together were strongest. This effect was even more pronounced upon re-seeding of surviving cells for spheroid formation and a second round of treatment, followed by the evaluation of surviving spheroidal-growing cells (Figure 3A, 2nd generation). Moreover, the measurement of aldehyde dehydrogenase isoform 1 (ALDH1) activity, which is defined as a marker for self-renewal capacity [28], confirmed that aspirin inhibited the potential for self-renewal (Figure 3B). These data suggest, that aspirin increases the therapeutic efficacy of gemcitabine by inhibition of inflammatory proteins and the self-renewal potential of CSCs.

### Aspirin eliminates patient-derived CSCs

**Figure 4:**
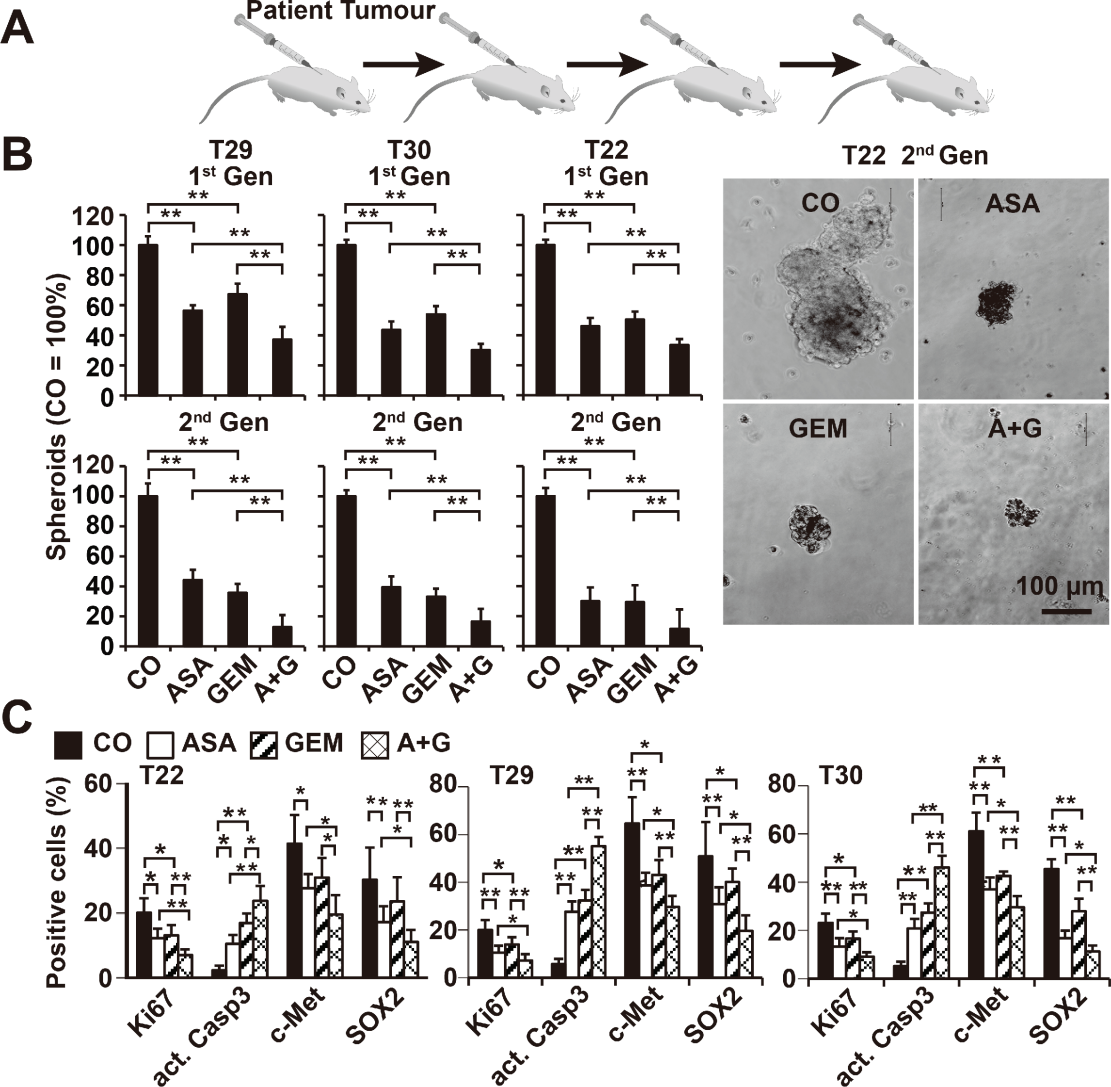
Aspirin inhibits the development of primary CSC spheroids and enhances gemcitabine efficacy **(A)** Primary pancreatic CSCs were selected by serial transplantation of freshly resected PDA tumor tissues (T29, T30, T22: compare Table 1) to immunodeficient mice, followed by *in vitro* culture on ultra-low attachment plates at a low density of 1–2 × 10^4^ cells/mL in serum-free, but growth factor-containing, medium for spheroid formation. **(B)** One week after culturing *in vitro*, the spheroids were treated as described. Five days later, the percentage of viable spheroids was determined (1st Gen). The number of spheroids in the control was set to 100%. Representative photographs from T22 spheroids 5 days after treatment at 100× magnification are shown at the right. The bar indicates 100 μm. **(C)** Cells from treated primary spheroids were collected on microscope slides by cytospin centrifugation. Expression of the proliferation marker Ki67, the apoptosis marker “cleaved, active fragment of caspase-3” (act. Casp3), and the CSC markers c-Met and SOX2 was examined by immunohistochemistry. The number of positive cells was quantified in 10 visual fields at 400× magnification, and the means ± SD are shown in the diagrams (**P < 0.01, *P < 0.05).

To extend these data, we used human primary CSC marker-enriched spheroidal-growing PDA cells isolated from resected PDA tissues from 3 different patients (T22, T29, and T30) (Table 1) followed by serial transplantation to mice (Figure 4A). At passage 6–12, the tumor cells were isolated from xenografts and cultured anchorage-independently *in vitro*, as we described previously [22, 29]. During this procedure, the highly aggressive cancer cells are enriched by clonal selection, which was confirmed by the enrichment of the CSC markers CD133 and c-Met from below 20% in the primary tumor to about 80% in the spheroidal cultures [22, 29]. The rational for this procedure was that a similar selection occurs in patient tumors: At the beginning of the disease most of tumors respond to chemotherapy, but after several cycles, the tumor acquires resistance, and this may be due to the clonal selection of the highly resistant cancer cells. The isolated primary CSCs were exposed to aspirin, gemcitabine, or both in combination. Five days later, the number of viable spheroids from the first generation (1^st^ Gen) was evaluated, followed by a second round of treatment (2^nd^ Gen). Whereas aspirin and gemcitabine alone reduced the percentage and size of the spheroids, combination treatment was significantly more effective and almost completely prevented the generation of secondary spheroids (Figure 4B). To evaluate the underlying signaling events, primary CSCs were collected on a microscope slide following cytospin centrifugation 72 h after treatment and then examined by immunohistochemistry and counting the percentage of positive cells. Aspirin and gemcitabine led to a significant reduction of proliferation, as indicated by labeling with Ki67-specific antibodies, expression of c-Met and SOX-2, and induction of the active form of caspase-3, indicating the induction of apoptosis (Figure 4C, Figure S3). These effects were observed with all treatments but were most significant for aspirin and gemcitabine in combination.

**Table 1:** Characteristics of patients and PDA tumor tissues

No.	Gender	Age	Grading	Pre-treatment
T22	m	79	pT, pT3, pN1 (5/31), G3, R1	not evaluated
T29	m	77	pT3, N1, G2, R2	not evaluated
T30	f	40	pT3, pN1 (16/30), G2, R1	not evaluated
5008	m	64	T3, pN1, (19/55), G2, L1, V1, Pn1	No
5009	f	51	pT3, pN1, G3, R0	No
5026	m	74	pT3, pN1, (8/33), G3, L1, V1	No
5027	f	60	pT3, pN1, (2/24), V1, Pn1, G2, R1	No
5031	m	60	pT3, pN1, (4/35), L1, V1, Pn1, G3, R1	No
5024	f	61	pT3, pN1, (27/37), L1, Pn1, G3, R1	No
4941	m	58	pT3, pN1, (9/21), Pn1, L1, G3, R1	ASA
4911	m	68	pT3, pN1, (10/22), G3, R1	ASA
4730	m	76	pT3, pN1, (5/22), L1, G2	ASA
4710	m	73	pT3, pN1, (2/35), L1, V0, PN1	ASA
4630	f	68	pT3, pN1, 82/25), G2, R0	ASA
4529	m	72	pT3, pN1, (4/61), L1, G3	ASA
4409	m	72	pT3, pN1, (9/18), G2	ASA
4945	m	63	ypT3, ypN0, (0/7), Pn1, R1	GEM
4912	m	54	ypT3, ypN1, yPn1, (1/18), R1	GEM
4827	m	56	ypT3, ypN0, (0/26), L1, Pn1, R1	GEM
4794	f	61	pT3, pN1, (1/10), G3, R1	GEM
4785	m	58	ypT3, ypN0, (0/15), R0	GEM
4318	m	63	ypT3, ypN0, (0/11), G0	ASA + GEM
4151	m	62	ypT3, ypN1, (1/23)	ASA + GEM
3738	f	71	ypT3, ypN0 (0/13)	ASA + GEM

f: Female; m: Male; IPMN: Intraductal papillary mucinous carcinoma; Tis: Carcinoma *in situ*, includes PanIN-3c; T1: Tumor limited to the pancreas, 2 cm or less in greatest dimension; T2: Tumor limited to the pancreas, more than 2 cm in greatest dimension; T3: Tumor extending beyond the pancreas; T4: Tumor involving the coeliac axis or the superior mesenteric artery; N0: No regional lymph node metastasis; N1: Regional lymph node metastasis; Borderline: Between benign and malignant; M0: No distant metastasis; M1: Distant metastasis; G1: Well-differentiated; G2: Moderately differentiated; G3: Poorly differentiated.

### Aspirin inhibits tumor growth, progression markers and desmoplasia *in vivo*

**Figure 5:**
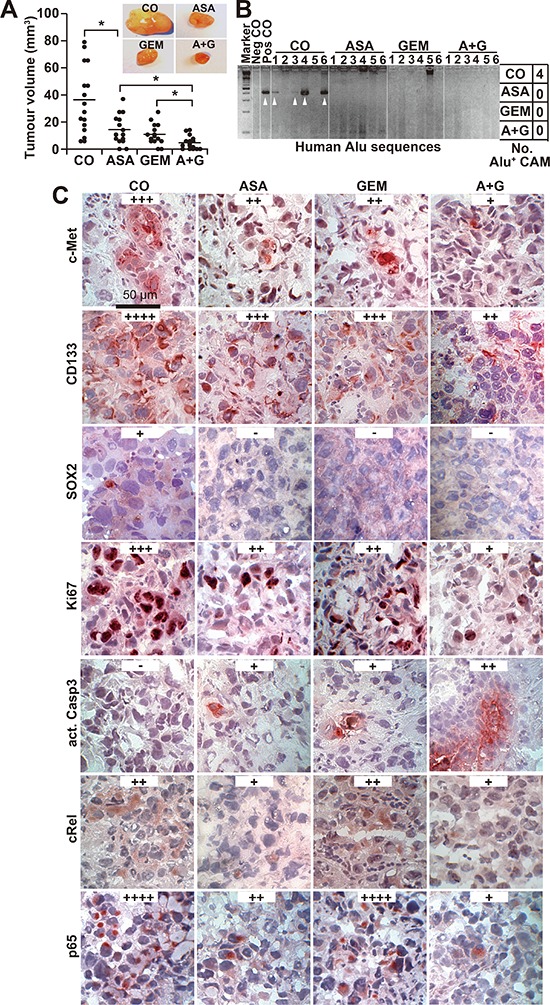
Aspirin inhibits tumor growth and invasion *in ovo* and enhances gemcitabine efficacy **(A)** PANC-1 cells were transplanted into a plastic ring placed on the CAM of 9-day-old chick embryos. Double-distilled water (CO, 40 μL), aspirin (ASA, 30 μL, 15 mM), gemcitabine (GEM, 10 μL, 100 nM) or aspirin and gemcitabine together (A + G) were placed on Whatman filter papers (0.5 cm^2^) directly adjacent to the tumor xenografts. Aspirin was applied at days 11, 13, 15 and 17 and gemcitabine at days 11 and 15. The tumor xenografts were resected at day 17, and the tumor volumes were measured with calipers. Representative images of each group are shown. The diagram shows single measurements and the means ± SD (**P < 0.01, *P < 0.05). **(B)** Genomic DNA was isolated from CAM tissue (*n* = 6) directly adjacent to the tumor xenografts, and a PCR with primers for human Alu sequences was performed. Double-distilled water served as a negative control (Neg CO), and genomic DNA isolated from a tumor xenograft served as a positive control (Pos CO). The DNA marker is shown in the first lane (Marker), and the table on the right summarizes the number of positive bands per group (No. Alu^+^ CAM). **(C)** Tumor tissue sections from xenografts were evaluated by immunohistochemistry for the expression of c-Met, CD133, Ki67, cleaved active fragment of caspase-3, c-Rel and p65. Representative images at 400× magnification are shown. The bar indicates 50 μm. Positive cells are colored red to dark-red. For evaluation of the expression levels, a semi-quantitative scoring system was used based on the percentage of positive cells: very high (++++), high (+++), medium (++), low (+) and absent (−).

The *in vivo* relevance of these data was examined by xenotransplantation of PANC-1 cells onto the CAM of fertilized chick eggs on developmental day 11 followed by *in ovo* treatment with aspirin on days 11, 13, 15 and 17 and gemcitabine treatment on days 11 and 15. The xenografts were resected on day 18. Whereas aspirin and gemcitabine alone significantly reduced the tumor volume, treatment with both in combination nearly completely prevented tumor formation (Figure 5A). To evaluate the invasion potential, genomic DNA was prepared from the CAM surrounding the tumor xenograft and from liver and lung tissue. Human Alu sequences, reflecting the presence of invading and metastasizing human cells, were detected by PCR. Whereas 4 of 6 CAM tissues from untreated eggs were Alu-positive, none of the tissues from the aspirin- or gemcitabine-treated groups were positive (Figure 5B). Alu sequences were not detectable in the liver or lung in any group, indicating that the xenografts did not spread to other organs (Figure S4A). In addition, we observed neither significant change in body weight of the embryos nor liver necrosis or developmental effects, indicating that the treatment was well tolerated (Figure S4B, S4C). Immunohistochemical staining for c-Met, CD133, SOX2, Ki67, cleaved fragment of caspase-3, p65 and c-Rel indicated that both single treatments reduced the proliferation and the expression of CSC markers and NF-κB subunits and induced apoptosis, although the combination treatment was most effective (Figure 5C); these findings were confirmed by immunofluorescence-double staining of the CSC markers EpCAM and Ki67 (Figure S4D).

**Figure 6:**
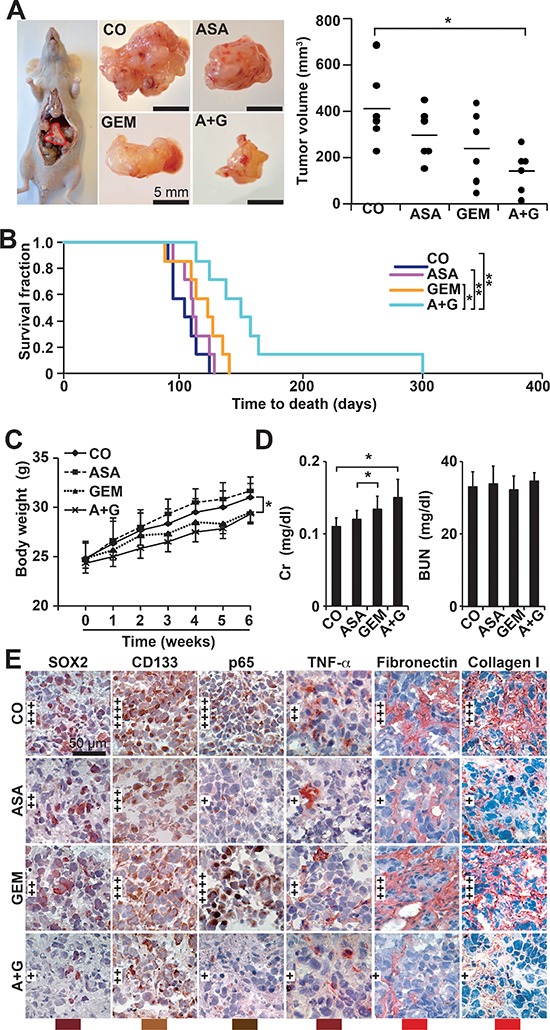
Aspirin inhibits tumor growth and progression of orthotopic mouse xenografts and enhances gemcitabine efficacy **(A)** A total of 1 × 10^6^ PANC-1 cells in 10 μL of PBS were injected into the pancreatic head of immunodeficient mice (Day 0), 12 mice per group. Aspirin was injected daily i.p. starting on day 1 (ASA, 200 mg/kg). Gemcitabine was injected once per week, beginning on day 7 (GEM, 12.5 mg/kg). Six mice of each group were euthanized 6 weeks after transplantation, followed by macroscopic inspection of metastases, tumor resection and measurement of tumor volumes using calipers. Representative images of tumor xenografts for each group are shown on the left, and a diagram containing the volumes of individual tumors and the means of each group is shown on the right (*P < 0.05, **P < 0.01). **(B)** The 6 residual mice of each group were observed until the time to death. For calculation of the survival fraction, the number of mice per group was set to 1 at day 0. **(C)** The mice were weighed weekly throughout the experiment, and the changes in body weight are shown. **(D)** Heparinized blood from mice was collected at the end of the experiment, and the levels of plasma creatinine (Cr) and blood urea nitrogen (BUN) were measured using a DRY-CHEM FCD3500. **(E)** Xenograft tumor tissue was analyzed by immunohistochemistry as described in Figure 5C.

To further confirm these data, we xenotransplanted PANC-1 cells orthotopically to the pancreas of immunodeficient mice followed by treatment with aspirin or gemcitabine. Aspirin was used in a dose of 200 mg/kg, according to a publication, in which the same concentration was successfully used for the treatment of mice with orthotopically growing PANC-1 xenografts [30]. The administration of aspirin or gemcitabine alone decreased tumor growth, but the combination of both agents significantly inhibited tumor formation (Figure 6A). These results are reflected by the survival time of mice of each group, which was significantly longer in the combination group (Figure 6B). Also, there was a significant longer survival of gemcitabine-treated mice upon co-treatment with aspirin. No macroscopic metastases were detected in the livers and lungs, but we observed two mice in the control group and one mouse in the gemcitabine group with celiac implantation metastasis, while no metastases were detected in the aspirin and combination groups (data not shown). While aspirin had no obvious side effects, as evident from measures of body weight, plasma creatinine and urea nitrogen, gemcitabine alone or combined with aspirin significantly reduced the body weight and increased the plasma creatinine levels (Figure 6C, 6D). In contrast, liver necrosis was not induced by any treatment (Figure S5). Immunohistochemical staining of xenograft tissue from treated mice revealed a reduction in SOX2, CD133, p65 and TNF-α, as well as the ECM components fibronectin and collagen, and more pronounced effects were observed following combination treatment (Figure 6E).

### Aspirin intake prior to surgery inhibits markers of tumor progression and collagen deposition in patient tissues

**Figure 7:**
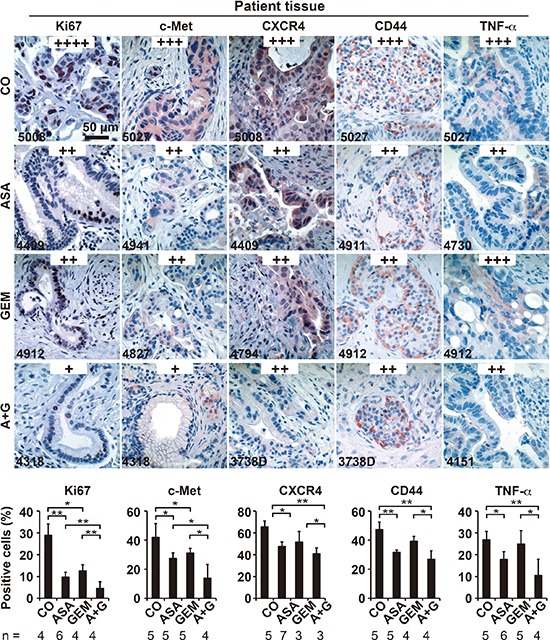
Aspirin intake before surgery inhibits the expression of progression markers in PDA patient tissue Tumor tissue sections derived from patients with documented pre-operative administration of aspirin (*n* = 7), gemcitabine (*n* = 5), aspirin plus gemcitabine (*n* = 3), or neither aspirin nor gemcitabine (*n* = 6) were evaluated by immunohistochemistry for the expression of the proliferation marker Ki67, the inflammatory factor TNF-α, and the CSC markers c-Met, CD44 and CXCR4. The scale bar indicates 50 μm. The number of positive cells was quantified in 10 visual fields at 400× magnification, and the means ± SD are shown in the diagrams. The data are presented as means ± SD (**P < 0.01, *P < 0.05).

**Figure 8:**
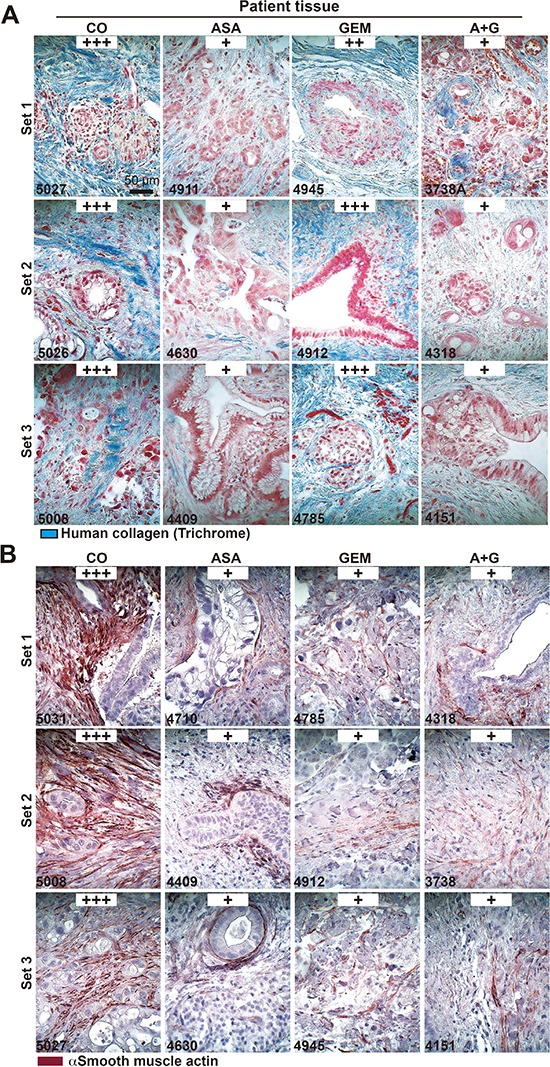
Aspirin intake before surgery reduces desmoplasia in patient tissues **(A)** The patient tissue described in Figure 6 was stained with trichrome to detect deposits of human collagen (blue) embedded between tumor cells (red). The scale bar indicates 50 μm. **(B)** Likewise, the above patient tissue was stained with an anti αSMA antibody for the detection of activated stellate cells (red). The scale bar indicates 50 μm. The intensity of collagen deposits or αSMA expression was evaluated as described in Figure 5C.

To examine the impact of aspirin in PDA patients, we examined tissues from patients that had taken aspirin (*n* = 5), gemcitabine (*n* = 4), both together (*n* = 3) or neither (*n* = 4) prior to resection (compare Table 1). In accordance with the former *in ovo* and mouse xenograft results, aspirin intake reduced the expression of Ki67, c-Met, CxCR4, CD44, and TNF-α (Figure 7). Because a recent multicenter retrospective study suggested a reduction in liver fibrosis mediated by aspirin [31], we examined the effect of aspirin on the deposition of collagen fibers in pancreatic tissues by trichrome staining and immunohistochemistry. Aspirin intake inhibited collagen deposition, whereas gemcitabine had no effect. However, the combination treatment resembled aspirin treatment alone, as observed in 3 representative sets of patient tissues (Figure 8A). Likewise, the expression of αSMA, which marks active, collagen-producing stellate cells, was strongly inhibited by both aspirin and gemcitabine, although no additional effect was observed with combination treatment (Figure 8B). These results strongly point to the direction, that aspirin may prevent progression of PDA by inhibition of the inflammatory response and thus the desmoplastic reaction.

## DISCUSSION

Considering the very poor prognosis of patients with PDA [1], more effective therapeutic strategies are required to achieve better outcomes. One major problem with PDA is the associated presence of extremely dense tumor stroma, which prevents the perfusion of therapeutics by chemo- and immunotherapy and may form a preventive niche for the highly aggressive CSC population [2]. Inflammation is known to drive this desmoplastic reaction, and enhanced activity of the pro-inflammatory signaling factor NF-κB contributes to the protection of pancreatic CSCs [3–6, 11]. Our current study demonstrated that aspirin inhibited CSC features, and this effect involved the inhibition of inflammatory activity, self-renewal potential, stem cell marker expression, tumor growth, metastasis and the stromal reaction.

### Aspirin has a preventive effect on the development of PDA

We observed that aspirin-treated PDA cells showed impaired potential for self-renewal due to the inhibition of spheroid and colony formation, ALDH1 activity, CSC marker expression and impaired tumor engraftment *in vivo*. In addition, daily administration of aspirin at a dose of 200 mg/kg for 5 weeks diminished the growth of human PDA tumor xenografts transplanted to the pancreas of immunodeficient mice. These results are in agreement with recent data obtained in genetically engineered mouse models of pancreatic cancer, which demonstrated that daily i.p. injection of 20 mg/kg aspirin delayed the progression of PanIN and cancer formation [19, 20]. Furthermore, 200 mg/kg aspirin administered daily delayed the growth of pancreatic cancer in an orthotopic mouse model using human PANC-1 xenografts [30]. The concentrations of aspirin administered to mice *in vivo* may be relevant for humans, as extrapolation of a 200 mg/kg aspirin mouse dose to a human dose, considering the surface area for humans [32], results in a daily aspirin dose of 16 mg/kg, or 960 mg aspirin for a 60 kg human, which is a dose that is used clinically for the treatment of inflammation and analgesia. Likewise, the aspirin concentrations used in our *in vitro* experiments correspond to peak plasma levels between 1–5 mM, which are measured in the serum of patients treated with aspirin for chronic inflammatory diseases [13, 33]. Our mouse data add important information to the growing body of evidence that aspirin indeed may be able to prevent the growth of PDA in patients, as indicated in a recently published case-control study [34]. In this study, data were obtained and evaluated for 362 patients with pancreatic cancer and 692 individuals without cancer from 30 hospitals in Connecticut between 2005 and 2009. A dose of 75 to 325 mg aspirin/day was administered for the prevention of heart disease but was found to reduce the risk of PDA by 48% after 3 years of treatment and by 60% after 20 years of treatment. Our findings are also consistent with the report that a low dosage of aspirin reduced colorectal tumor genesis by 60% among 311 patients with colorectal adenomas and adenocarcinomas [35]. However, in contrast to our study, none of these studies addressed the efficacy of aspirin toward highly aggressive, CSC-like PDA cells.

### Aspirin primarily targets highly aggressive cells and is well tolerated

To determine whether aspirin primarily affects more aggressive cells, we used different strategies. First we used a set of established PDA cell lines with documented low, median or high aggressiveness and found that aspirin mainly affected the more aggressive and gemcitabine-resistant cells. Second, we demonstrated that aspirin strongly reduces the self-renewal potential of established and primary CSC cells by inhibition of colony- and spheroid formation and ALDH1 activity. Our data suggest that aspirin eliminates pancreatic CSCs. Similar results were recently found in colorectal cancer [36]. In the latter study, Moon and colleagues demonstrated that nonsteroidal anti-inflammatory drugs, including indomethasin, sulindac, aspirin, celecoxib, γ-secretase inhibitor and PPARG antagonist significantly decreased the number of colosphere formation. In addition, indomethacin enhanced the efficacy of 5-FU in mouse tumor xenografts [36], which is in line with our *in vivo* data, where we demonstrate that aspirin enhances the efficacy of gemcitabine in chick or orthotopic mouse xenografts. Moreover, a study performed by Deng and colleagues [37] found that celecoxib downregulates the expression of the CSC marker CD133 through inhibition of the Wnt signaling pathway in colon cancer cells. This finding supports our data of aspirin-mediated downregulation of several CSC markers in PDA. To confirm our observation that aspirin mainly attacks the highly aggressive pancreatic cancer cells, we examined the effect of aspirin on non-malignant pancreatic duct cells and mesenchymal stem cells. We found that aspirin exhibited no cytotoxicity to these primary cells at a therapeutically active concentration of 5 mM *in vitro*. Likewise, aspirin did not induce adverse side effects in chick embryos and mice. Although aspirin is known to increase the risk of gastrointestinal bleeding, this type of side effect may be tolerable, since it can be prevented with proton pump inhibitors in patients [38]. More importantly, long-time daily aspirin use was shown to reduce all-cause mortality, including fatal bleeding mortality, by approximately 10% [17]. Given that cancer-related death is more serious than non-fatal bleeding complications, aspirin indeed may hold great potential benefits for both cancer prevention and treatment.

### Aspirin enhances the therapeutic efficacy of gemcitabine

We proceeded to evaluate the effect of aspirin on the therapeutic efficacy of gemcitabine, which has been suggested to enrich the therapy-resistant CSC population [39], resulting in progression and metastasis. Our results demonstrated that aspirin enhanced the ability of gemcitabine to reduce cell viability and the potential for self-renewal and to induce apoptosis. These data were further supported by *in vivo* findings showing that aspirin led to complete inhibition of tumor growth, engraftment, invasion and metastasis when administered in conjunction with gemcitabine. We assume that this result may be partly due to the observed aspirin-mediated inhibition of TNF-α expression and NF-κB activity, which is induced by gemcitabine in highly aggressive CSC-like cells. This observation emphasizes the suggested relation between inflammation and PDA. Indeed, almost all risk factors for PDA, such as pancreatitis, are associated with chronic inflammation [40], and activation of NF-κB and TNF-α is crucial for the development of pancreatitis [41]. These events may be prevented by treatment with aspirin, which thereby may reduce the risk of developing PDA or the progression of existing PDA. Previous studies support this notion and demonstrate that inactivation of the NF-κB pathway effectively prevents the formation of human orthotopically transplanted PDA xenografts in mice [30] and the formation of inflammation/colitis-associated cancer in a mouse model [42]. According to the literature and our own observations, highly gemcitabine-resistant, aggressive PDA cells possess higher NF-κB activity compared to more sensitive PDA cells [43], reflecting the role of NF-κB in conferring CSC features [11]. This scenario emphasizes the notion that gemcitabine treatment leads to enrichment of CSCs and thus progression [39], which is prevented by aspirin co-treatment.

### Aspirin reduces deposition of the ECM

The results obtained from human PDA xenografts in mice as well as tissues from patients who had or had not taken aspirin before surgery suggest that aspirin strongly reduces the deposition of ECM components, such as fibronectin and collagen, and also leads to a reduction of αSMA-positive stellate cells. The effect of aspirin on ECM synthesis in PDA has to our knowledge not been studied. However, Miltyk and colleagues demonstrated in 1996 that aspirin evokes a decrease in collagen synthesis in cultured fibroblasts [44]. A more recent report from 2004 suggested that the inhibitory effect of aspirin on collagen biosynthesis in fibroblasts is coupled to the inhibition, but not expression, of prolidase phosphorylation and to the down-regulation of the intracellular signal transmitted by the β_1_-integrin receptor [45]. Likewise, nickel-induced collagen biosynthesis in human fibroblasts, which may be responsible for pulmonary fibrosis upon exposure to nickel compounds by inhalation, was prevented by aspirin [46]. The obvious clinical relevance of these findings has recently been confirmed by a multicenter retrospective study of recurrent hepatitis C after liver transplantation [31]. The authors came to the conclusion that low-dose aspirin of 75 or 100 mg, given for hepatic artery thrombosis after liver transplantation, reduced the progression of liver fibrosis [31].

## CONCLUSION

The present study highlights the suggested preventive and therapeutic effects of aspirin in PDA by demonstrating the elimination of CSC features, inflammatory factors and desmoplasia. Because we observed no side effects with aspirin, our data further support the potential for aspirin to be used in the prevention of PDA, as well as an adjuvant agent to improve the efficacy of chemotherapy in patients suffering from PDA.

## MATERIALS AND METHODS

### Human primary and established PDA cell lines

The established human PDA cell lines PANC-1, AsPC-1, and BxPc-3 and the human hTERT-HPNE immortalized pancreatic ductal cell line CRL-4023 were obtained from the American Type Culture Collection (ATCC) and cultured in DMEM (18 mmol/L glucose) supplemented with 10% FCS and 5% HEPES or in ATCC complete growth medium, respectively. Gemcitabine-resistant BxPc-3 cells (BxPc-3/GEM) have been described [11]. Human primary mesenchymal stem cells (MSCs) and the primary PDA cell lines PaCaDD183, PaCaDD159 and PaCaDD135 were isolated and cultured as previously described [11, 47]. All cells were grown in a humidified incubator at 37°C and 5% CO_2_ and authenticated throughout the culture by their typical morphology. The established cell lines were recently authenticated by a commercial service (Multiplexion, Heidelberg, Germany). Mycoplasma-negative cultures were ensured through monthly mycoplasma tests.

### Reagents

Aspirin (> 99% pure, Sigma-Aldrich, Steinheim, Germany) was dissolved in ddH_2_O to prepare a 0.5 M, pH 7.0 stock solution and stored in aliquots at −20°C. Each stock solution was used only once immediately after thawing. Gemcitabine solution (Eli Lilly, Indianapolis, IN, USA) was diluted in cell culture medium to prepare a 100 μM stock solution, and aliquots were stored at −20°C. The final concentrations of the solvents in the media were 0.1% or less.

### Selection of CSC-enriched spheroidal cells from patient tumors

Primary CSCs were isolated from surgical non-diagnostic specimens by serial transplantation to mice followed by *in vitro* spheroidal culture, as described previously [22]. Patient materials were obtained under the approval of the ethical committee of the University of Heidelberg after receiving written informed consent from the patients. The diagnoses were established by conventional clinical and histological criteria according to the World Health Organization (WHO). All surgical resections were indicated by the principles and practice of oncological therapy.

### Cell viability assay

Cell viability was measured using 3-(4,5- dimethylthiazol-2-yl)-2,5-diphenyltetrazolium bromide (MTT), as described previously [11].

### AnnexinV staining and flow cytometry analysis of apoptosis

Cells were stained with FITC-conjugated AnnexinV (BD Biosciences, Heidelberg, Germany) after respective treatments and analysed by flow cytometry, as described previously [11].

### ALDH1 activity

ALDEFLUOR substrate (5 μL; Aldagen, Inc., Durham, NC, USA) was added to 1 × 10^6^ treated tumour cells, and ALDH1 activity was measured by flow cytometry analysis according to the manufacturer's instructions.

### Electrophoretic mobility shift assay (EMSA)

Nuclear protein extracts were prepared using NE-PER® Nuclear and Cytoplasmic Extraction Reagents, and the bandshift reaction was performed using the Light Shift® Chemiluminescent EMSA Kit, according to the manufacturer's instructions (Thermo Scientific), and biotin 3´end-labeled oligonucleotides or unlabelled oligonucleotides (MWG Biotech), as described previously [22].

### Colony-forming assay

Forty-eight hours after treatment, the cells were re-seeded in complete medium in 6-well tissue culture plates. Colonies were evaluated 14 days later, as described previously [11].

### Spheroid assay

Tumor cells were cultured in human NeuroCult NS-A serum-free medium with supplements (Stem Cell Technologies, Inc.), and analysis of spheroid formation was performed as described previously [11].

### Western blot analysis

Whole-cell protein extracts were prepared using a standard protocol, and proteins were detected by Western blot analysis as described previously [11]. Mouse monoclonal antibodies directed against human p52 (Santa Cruz Biotechnology, Santa Cruz, CA, USA), TNF-α (Abcam, Cambridge, UK), and β-Actin (Sigma-Aldrich, St. Louis, MO, USA) were used. Rabbit polyclonal antibodies against human Nanog, SOX2, (Abcam, Cambridge, UK), Oct4 (Cell Signaling, Danvers, MA, USA), c-Rel, and p65 (Santa Cruz Biotechnology, Santa Cruz, CA, USA) were used.

### Transplantation of tumor cells to the chorioallantoic membrane (CAM) of fertilized chick eggs

Fertilized eggs from genetically identical hybrid Lohman Brown (LB) chicks were obtained from a local ecological hatchery (Geflügelzucht Hockenberger, Eppingen, Germany). Tumor cells were transplanted to the CAM, followed by treatment and evaluation of tumor take and volume, as described previously [22].

### Immunohistochemistry and immunofluorescence staining

Staining was performed on 6-μm frozen or paraffin-embedded tissue sections or on primary spheroidal cultures that were transferred to glass slides by cytospin centrifugation, as described previously [22, 29]. The primary antibodies were: Rabbit pAbs against human c-Met, CD133, Sox2 (Abcam, Cambridge, UK), CD44 (GeneTex Inc., USA); Ki67 (Thermo Scientific, Rockford, IL, USA); the cleaved fragment of activated human caspase-3 (R&D Systems, Abingdon, UK); c-Rel (Santa Cruz Biotechnology Inc., Germany); CxCR4 (GeneTex Inc., USA); Fibronectin and Collagen type I (Acris Antibodies GmbH, Herford, Germany); Rabbit mAb against TNF-α and p65 (Rel A, Cell Signalling Danvers, MA, USA); Mouse mAb against TNF-α (Cell Signaling) and α-smooth muscle actin (αSMA, α-Actin clone 1A4, Santa Cruz, Heidelberg, Germany).

### Extraction of genomic DNA and human Alu PCR amplification

Genomic DNA was isolated from freshly harvested chick tissue using the DNeasy Blood and Tissue Kit (Qiagen, Hilden, Germany). PCR was performed as described previously [22].

### Nude mice and xenografts

PANC-1 cells (1 × 10^6^ cells in 10 μL PBS) were injected into the subcapsular region near the head of the pancreas of 6-week-old NMRI (nu/nu) male mice (day 0) through an abdominal midline incision with a fine needle after the mouse was placed under general anesthesia. To avoid possible leakage of tumor cells, the injection was performed using a Leica M651 microscope (Leica, Wetzlar, Germany). Mice carrying PANC-1 xenografts were randomly divided into 4 groups of 12 animals each, which received the following intraperitoneal (i.p.) injections starting at day 7 after tumor transplantation: Control group: H_2_O daily; Aspirin group: 200 mg/kg daily; Gemcitabine group: 12.5 mg/kg once per week; Combination group: 200 mg aspirin/kg daily and 12.5 mg gemcitabine/kg once per week. Six mice of each group were sacrificed at week 6, and tumor volumes (V) were determined using two diameters and were calculated using the following formula: V = 1/2 (length × width^2^). The residual 6 mice of each group were observed and the time to death was documented. The animals were euthanized in case of ascites formation or other signs of suffering. Animal experiments were performed in the animal facilities of the University of Heidelberg after receiving approval from the authorities (Regierungspräsidium Karlsruhe, Germany).

### Statistical analysis

Data obtained with established and primary cell lines are presented as the means ± SD from at least three separate experiments, each performed in triplicate. The experiments with primary spheroidal cells were performed twice in triplicate due to the limitation of patient-derived, primary spheroids. The significance of the data was analyzed using Student's *t*-test for parametric data and the Mann-Whitney test with Bonferroni corrections for nonparametric data. *P* < 0.05 was considered statistically significant (**P < 0.01, *P < 0.05).

## SUPPLEMENTARY FIGURES



## Abbreviations

ALDH1: Aldehyde dehydrogenase 1
CSCs: Cancer stem cells
CAM: Chorioallantoic membrane
EMTE: pithelial mesenchymal transition
gemcitabine: Gemcitabine
EMSA: Electrophoretic mobility shift assay
PDA: Pancreatic ductal adenocarcinoma
