# Correction: Aspirin counteracts cancer stem cell features, desmoplasia and gemcitabine resistance in pancreatic cancer

**DOI:** 10.18632/oncotarget.28527

**Published:** 2024-07-17

**Authors:** Yiyao Zhang, Li Liu, Pei Fan, Nathalie Bauer, Jury Gladkich, Eduard Ryschich, Alexandr V. Bazhin, Nathalia A. Giese, Oliver Strobel, Thilo Hackert, Ulf Hinz, Wolfgang Gross, Franco Fortunato, Ingrid Herr

**Affiliations:** ^1^Molecular OncoSurgery, University of Heidelberg and German Cancer Research Center (DKFZ), Heidelberg, Germany; ^2^Section Surgical Research, University of Heidelberg, Heidelberg, Germany; ^3^Department of General, Visceral and Transplantation Surgery, University of Heidelberg, Heidelberg, Germany; ^4^Gastrointestinal Surgery, Zhongshan Hospital of Xiamen University, Xiamen, China

**This article has been corrected:** In Figure 7, images for the control groups were accidentally inserted into the ASA, GEM, and A+G rows, instead of those from the treatment groups. In addition, in Supplementary Figure 3, panel T22, the images for samples act. Casp3 and c-Met are accidental duplicates. The corrected Figure 7 and Supplementary Figure 3, produced using the original data, are shown below. The authors declare that these corrections do not change the results or conclusions of this paper.

Original article: Oncotarget. 2015; 6:9999–10015. 9999-10015. https://doi.org/10.18632/oncotarget.3171

**Figure 7:**
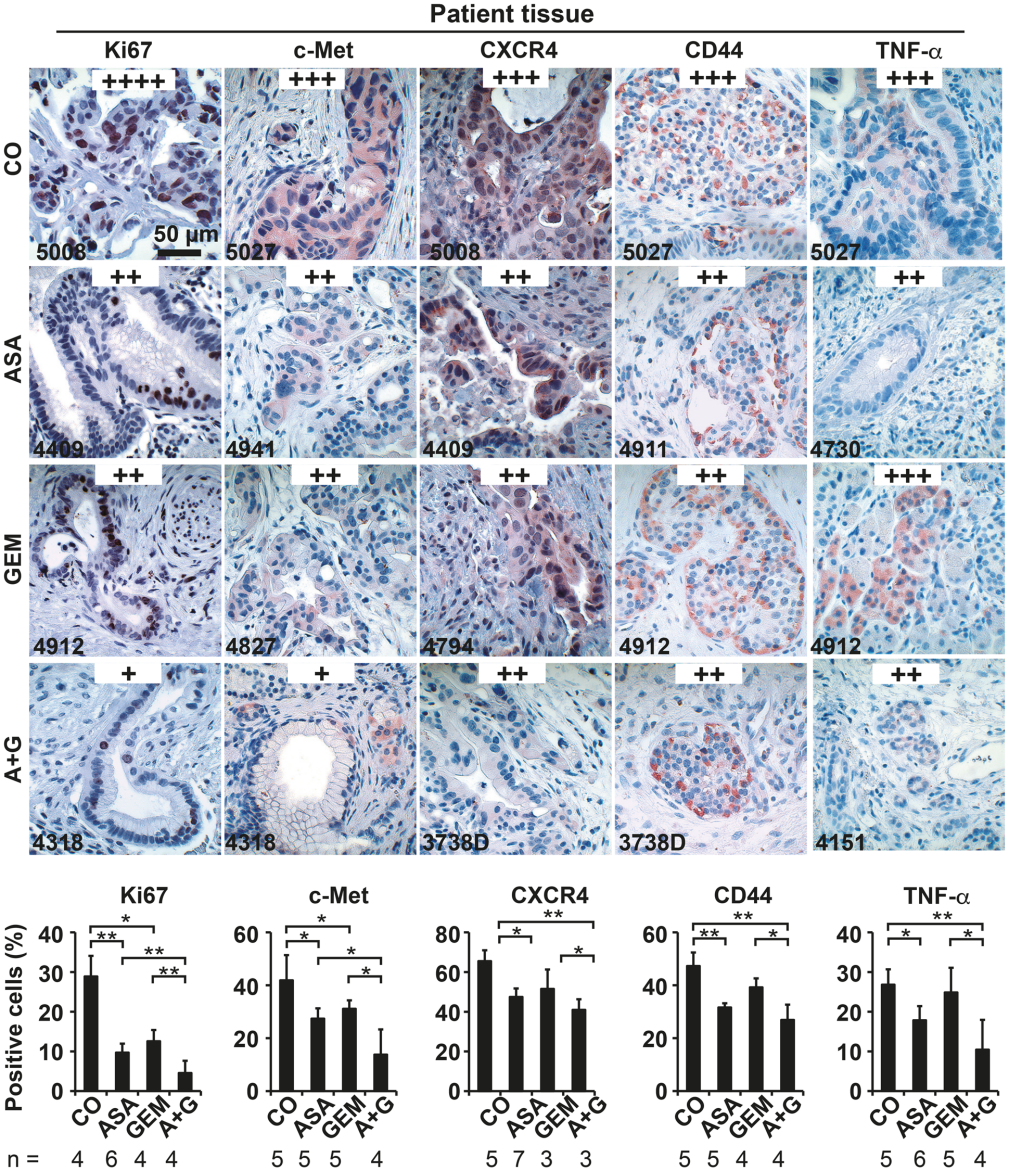
Aspirin intake before surgery inhibits the expression of progression markers in PDA patient tissue. Tumor tissue sections derived from patients with documented pre-operative administration of aspirin (*n* = 7), gemcitabine (*n* = 5), aspirin plus gemcitabine (*n* = 3), or neither aspirin nor gemcitabine (*n* = 6) were evaluated by immunohistochemistry for the expression of the proliferation marker Ki67, the inflammatory factor TNF-α, and the CSC markers c-Met, CD44 and CXCR4. The scale bar indicates 50 μm. The number of positive cells was quantified in 10 visual fields at 400× magnification, and the means ± SD are shown in the diagrams. The data are presented as means ± SD (^**^ *P* < 0.01, ^*^ *P* < 0.05).

**Supplementary Figure 3:**
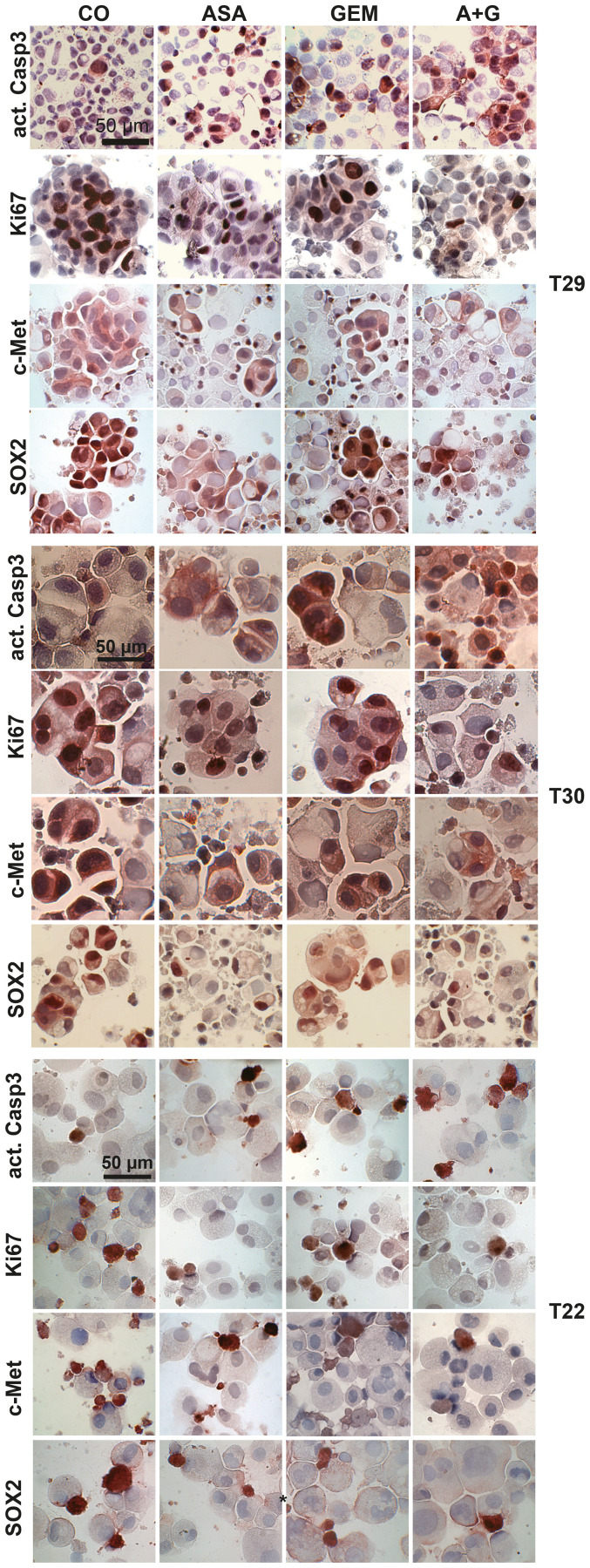
Aspirin inhibits the expression of progression markers in primary CSCs spheroids and enhances gemcitabine efficacy. Immunohistochemical staining is shown for primary spheroids, which were obtained and treated as described in Figure 4.

