# Supplementary material for: Aspirin counteracts cancer stem cell features, desmoplasia and gemcitabine resistance in pancreatic cancer
Source: Oncotarget. 2015 Feb 5;6(12):9999–10015. doi: 10.18632/oncotarget.3171 (PMC4496413; doi:10.18632/oncotarget.3171)
Supplement: Supplementary file 1 [file oncotarget-06-9999-s001.pdf]

SUPPLEMENTARY FIGURES

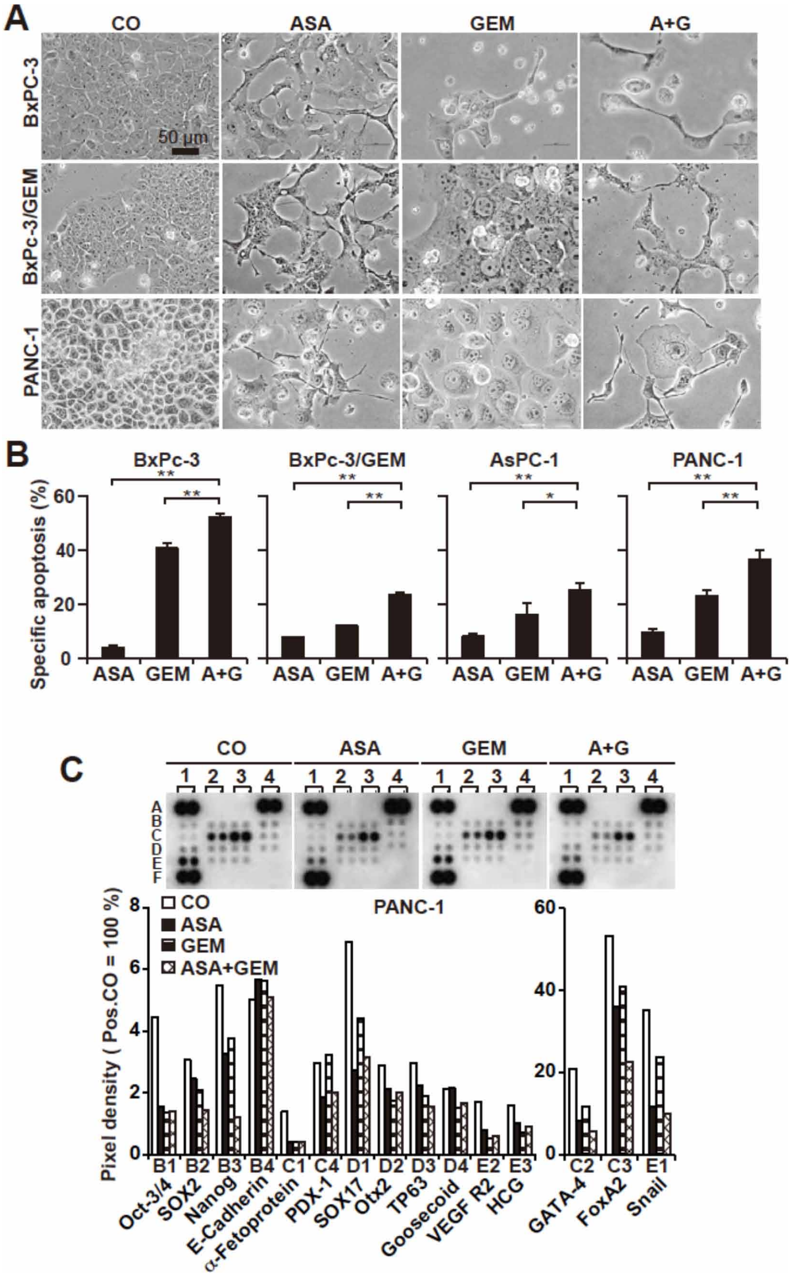

Supplementary Figure S1: Aspirin overcomes gemcitabine resistance and alters the expression of reprogramming factors. **(A)** BxPc-3, BxPc-3/GEM, and PANC-1 cells were treated as described in Figure 1A, and 72 h later, the morphology was documented by microscopy at 200× magnification. Representative pictures are shown, and the bar indicates 50 µm. **(B)** Cells were treated as described above, and after 72 h, induction of apoptosis was evaluated by staining with AnnexinV-FITC followed by flow cytometry. The percentage of specific apoptosis was calculated as follows: 100 × [(experimental apoptosis (%) – spontaneous apoptosis of control (%))/(100 – spontaneous apoptosis of control (%)]. **(C)** Proteins from PANC-1 cells were isolated 48 h after treatment and incubated with the nitrocellulose membranes of an antibody array kit for the detection of human pluripotent stem cell markers. The binding of proteins to antibodies spotted on the membrane was detected using biotinylated secondary antibodies, streptavidin-HRP and chemiluminescence (upper pictures). The pixel density was quantified using the ImageJ software and normalised to the mean pixel intensity of reference spots located at the coordinates A1, A4 and F1 on the membrane. Spot E4 is the negative control, where PBS, instead of the antibody, was spotted onto the membrane. This experiment was performed once in duplicate, and the mean values are shown.

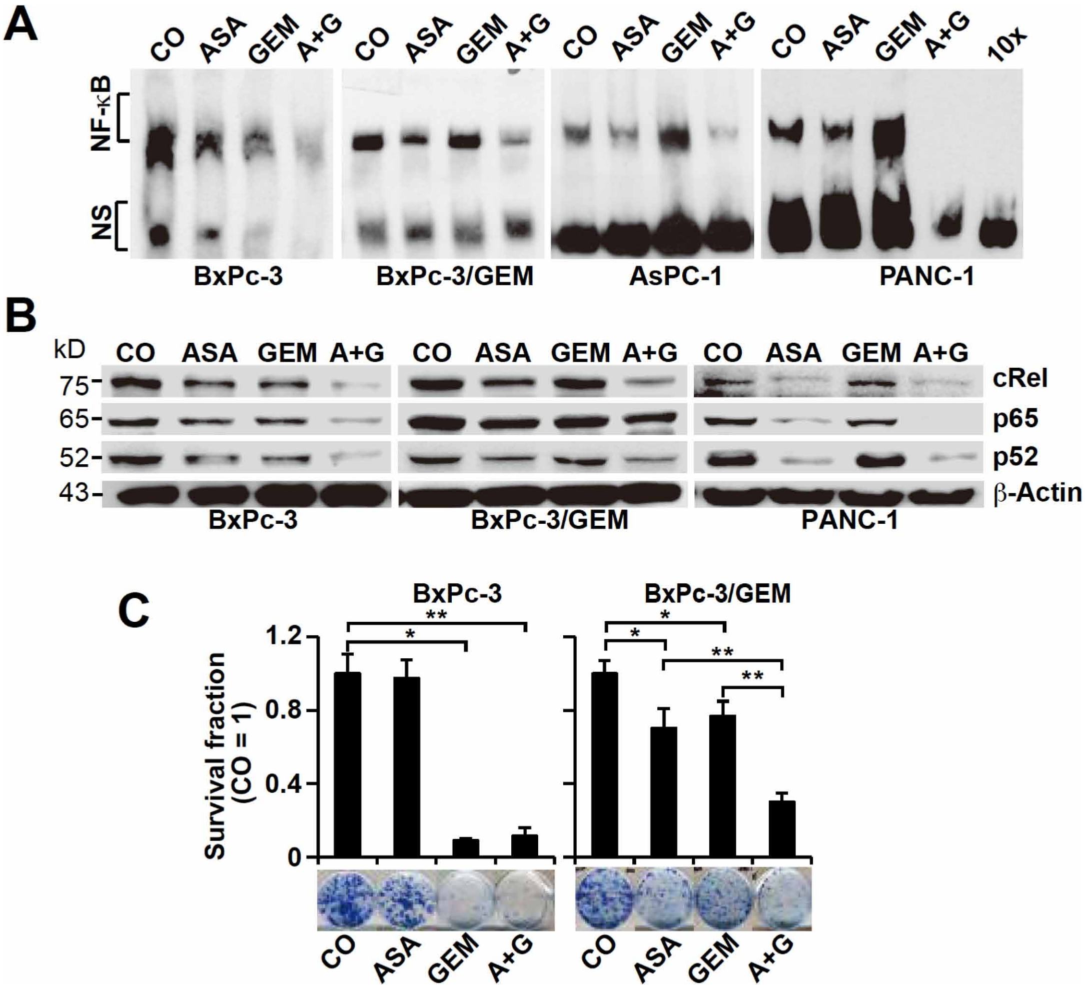

Supplementary Figure S2: Aspirin inhibits colony formation and enhances gemcitabine efficacy. **(A)** BxPc-3, BxPc-3/GEM, AsPC-1 and PANC-1 cells were treated as described, and after 24 h, nuclear extracts were prepared and DNA binding was analysed by EMSA using a specific biotin-labelled oligonucleotide probe for NF-κB. Specific NF-κB shifts (NF-κB) and non-specific shifts (NS) are marked on the left. Competition with a 10-fold excess of unlabelled oligonucleotide (10x) served as a control for the specificity of binding. **(B)** Likewise, 48 h after treatment, expression of the NF-κB subunits c-Rel, p65 and p52 was analysed by Western blot. **(C)** The colony-forming capacity and the survival fraction were determined in BxPc-3 and BxPc-3/GEM cells as described in Figure 2A. (BxPc-3: 3,000 cells/well; BxPc-3/GEM: 2,000 cells/well). The data are presented as means ± SD (**$P < 0.01$, *$P < 0.05$).

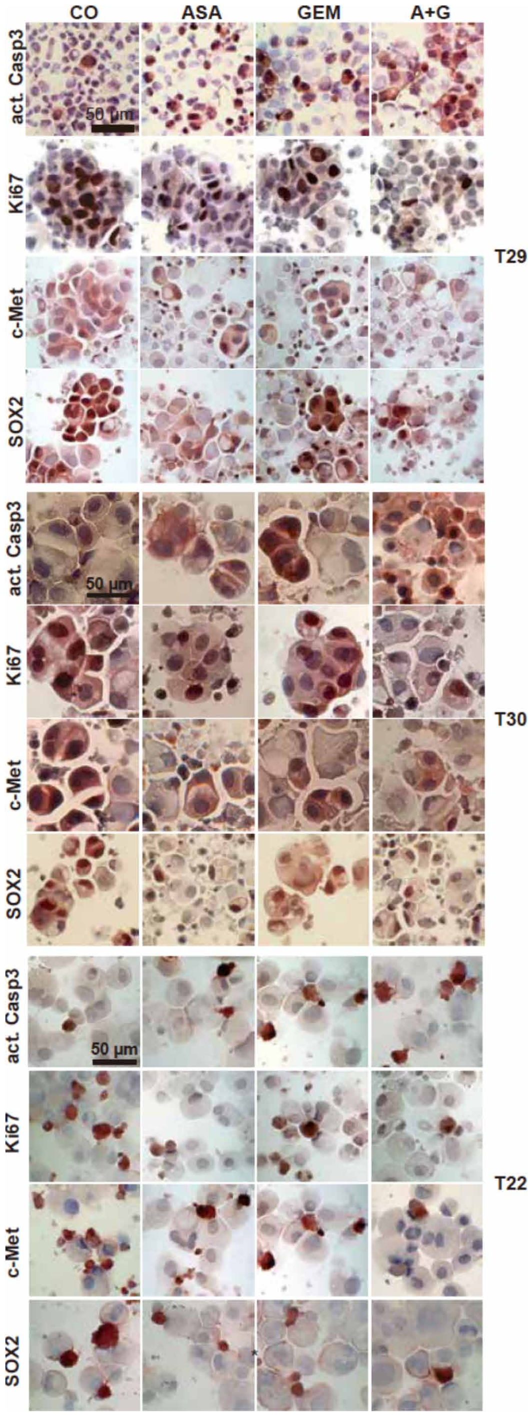

Supplementary Figure S3: Aspirin inhibits the expression of progression markers in primary CSCs spheroids and enhances gemcitabine efficacy. Immunohistochemical staining is shown for primary spheroids, which were obtained and treated as described in Figure 4.

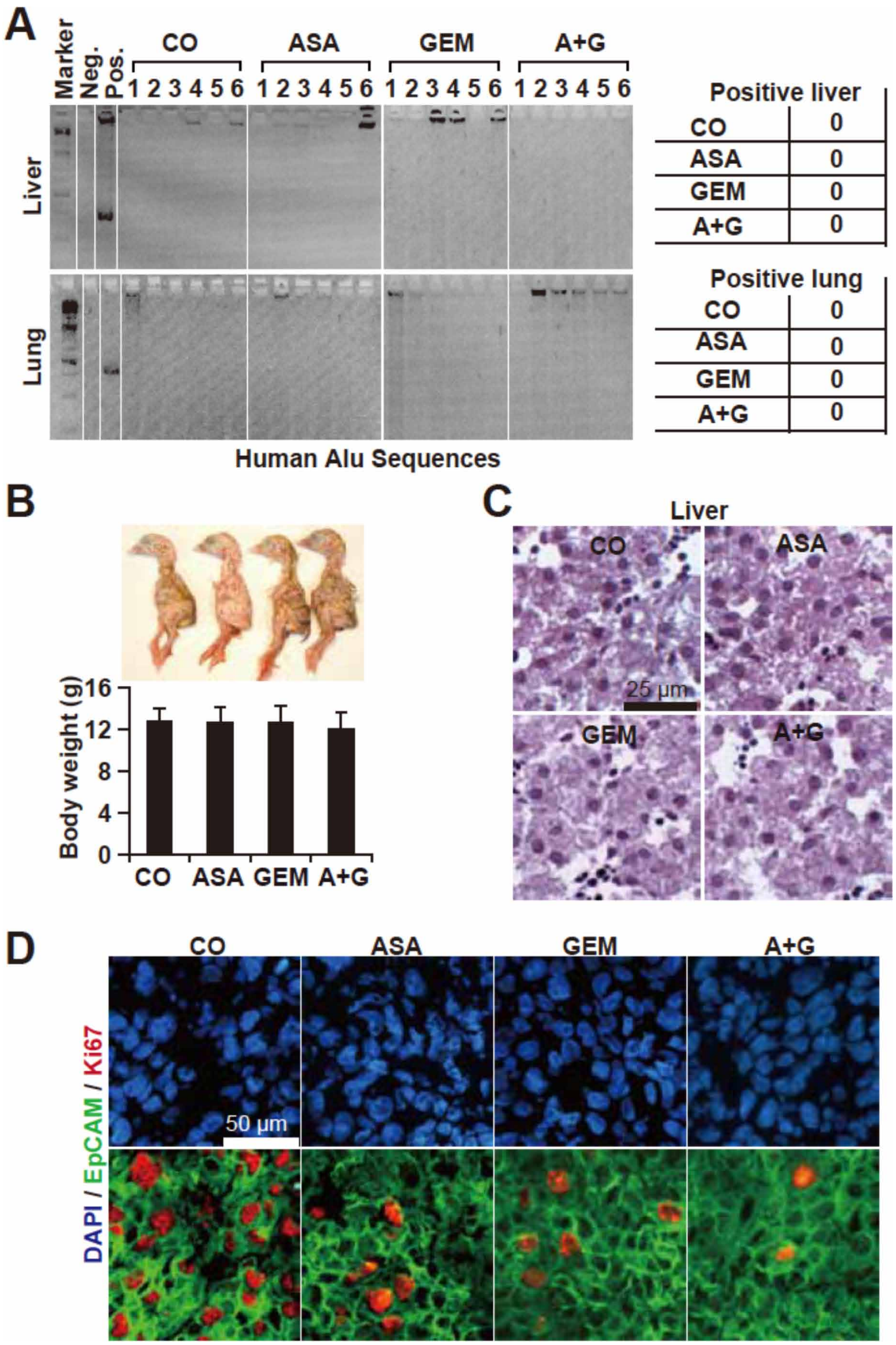

Supplementary Figure S4: Aspirin inhibits tumour growth and invasion *in ovo* and enhances gemcitabine efficacy. (A) Genomic DNA was isolated from the liver and lung tissues of embryos from each group of xenotransplanted eggs ($n = 6$), and an Alu PCR was performed as described in Figure 4B. **(B)** The weight of the embryos in each group was determined at day 17, and the result is presented as the mean weight ± SD. **(C)** Representative haematoxylin and eosin (H&E) staining of embryonic liver tissues is shown. The bar indicates 25 µm. **(D)** Xenograft tumour tissue sections were stained with human-specific antibodies to EpCAM (green) and Ki67 (red), and the nuclei were visualised by DAPI staining (blue). Representative photographs at 400× magnification) are shown, and the bar indicates 50 µm.

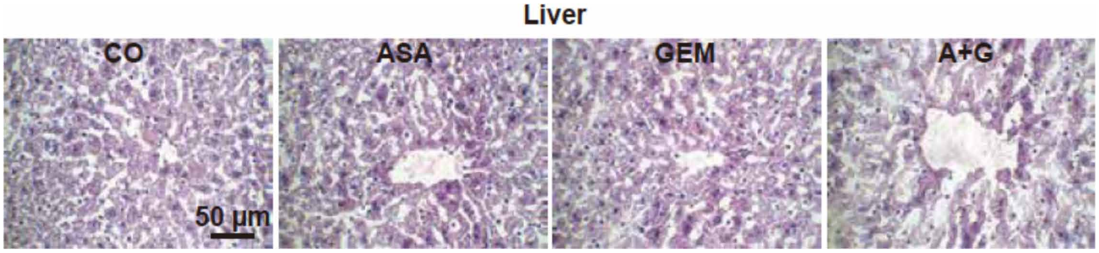

Supplementary Figure S5: Aspirin and gemcitabine do not induce liver necrosis in mice. Representative H&E stains of mouse liver tissue derived from mice with orthotopically transplanted PANC-1 xenografts, as described in Figure 6A. The scale bar indicates 50 µm.
